# Automatic pelvic fracture segmentation: a deep learning approach and benchmark dataset

**DOI:** 10.3389/fmed.2025.1511487

**Published:** 2025-04-15

**Authors:** Yanzhen Liu, Sutuke Yibulayimu, Gang Zhu, Chao Shi, Chendi Liang, Chunpeng Zhao, Xinbao Wu, Yudi Sang, Yu Wang

**Affiliations:** ^1^Beijing Advanced Innovation Center for Biomedical Engineering, School of Biological Science and Medical Engineering, Beihang University, Beijing, China; ^2^Beijing Rossum Robot Technology Co., Ltd., Beijing, China; ^3^Department of Orthopaedics and Traumatology, Beijing Jishuitan Hospital, Beijing, China

**Keywords:** CT segmentation, deep learning, pelvic fracture, reduction planning, image-guided surgery

## Abstract

**Introduction:**

Accurate segmentation of pelvic fractures from computed tomography (CT) is crucial for trauma diagnosis and image-guided reduction surgery. The traditional manual slice-by-slice segmentation by surgeons is time-consuming, experience-dependent, and error-prone. The complex anatomy of the pelvic bone, the diversity of fracture types, and the variability in fracture surface appearances pose significant challenges to automated solutions.

**Methods:**

We propose an automatic pelvic fracture segmentation method based on deep learning, which effectively isolates hipbone and sacrum fragments from fractured pelvic CT. The method employs two sequential networks: an anatomical segmentation network for extracting hipbones and sacrum from CT images, followed by a fracture segmentation network that isolates the main and minor fragments within each bone region. We propose a distance-weighted loss to guide the fracture segmentation network's attention on the fracture surface. Additionally, multi-scale deep supervision and smooth transition strategies are incorporated to enhance overall performance.

**Results:**

Tested on a curated dataset of 150 CTs, which we have made publicly available, our method achieves an average Dice coefficient of 0.986 and an average symmetric surface distance of 0.234 mm.

**Discussion:**

The method outperformed traditional max-flow and a transformer-based method, demonstrating its effectiveness in handling complex fracture.

## 1 Introduction

Pelvic fractures are classified as one of the most severe forms of orthopedic injury, typically resulting from high-energy trauma. A study involving 11,149 patients has demonstrated that pelvic fracture leads to a mortality of 14.2%, significantly higher than other types of injuries (1). The anatomical complexity of the pelvic ring involves numerous muscle groups, ligaments, neurovascular bundles, and other soft tissues, making its surgical intervention the most challenging one and posing significant treatment obstacles (2).

The goal of surgical management for pelvic fractures is to restore the bone's original anatomy to regain lost functional mobility. This process is categorized into open reduction and closed reduction surgeries. Open reduction surgery often necessitates extensive dissection, leading to considerable tissue damage and an elevated risk of complications. In contrast, closed reduction surgery is desired for its minimally invasive nature and hence the reduced recovery time (3). In recent years, the exploration and clinical implementation of robotic-assisted closed fracture reduction surgery have significantly enhanced the accuracy of fracture reduction while minimizing radiation exposure for both patients and surgeons (4). Regardless of whether manual or robotic-assisted reduction is employed, segmentation of the fractures from preoperative computed tomography (CT) is crucial. This step is fundamental for trauma diagnosis and reduction planning, aiming to identify and determine the optimal anatomical reduction position to restore the natural state of the pelvic bone.

Conventionally, semi-automated approaches are employed to delineate the anatomy of pelvic fractures. The initial steps involve thresholding and region-growing techniques to extract bone regions by adjusting the threshold and precisely locating seed points (5). Subsequently, the fracture surface is manually outlined, either by refining segments in a 3D view or by editing the segmentation masks on a slice-by-slice basis. This labor-intensive process often takes more than 30 minutes, especially when fracture fragments are intertwined or partially attached (6). Furthermore, the complexity and the induced variability of pelvic fractures mean that manual segmentation relies heavily on the clinician's experience, highlighting a pressing need for an automated solution for segmenting pelvic fracture fragments from CT images.

Deep learning has been successfully applied to various bone segmentation tasks, demonstrating its effectiveness (7). Nevertheless, learning-based methods specifically addressing pelvic fracture segmentation remain under-explored. Several factors in image characteristics contribute to this challenge:

- The intricate anatomy of the fractured pelvis, combined with surrounding bones such as sacralized lumbar vertebra, fractured vertebra or femur, and the potential presence of patient's hands during CT scanning, complicates the differentiation of pelvic bones.
- Unlike the more prevalent organ segmentation tasks, where models can often intuitively grasp the typical shape of an object, discerning the shape of bone fragments is more complex due to the significant variation in fracture types and morphologies (8).
- The actual fracture surface is diverse in its presentation. It can manifest as a vast space when fragments are isolated and displaced, a minor gap when fragments are isolated but stable, a crease when fragments are not fully separated, compression when fragments collide, or a blend of these scenarios. This diversity results in quite different image intensity profiles around the fracture site.
- The inconsistency in the number of bone fragments present in pelvic fractures poses a challenge in establishing a uniform labeling approach suitable for every fracture type and case.

In this study, we propose a deep learning-based method to segment pelvic fracture fragments from preoperative CT images. Our major contributions are threefold:

- We proposed a completely automated pipeline for pelvic fracture segmentation, which is the first attempt to apply deep learning to this task to the best of our knowledge.
- We designed a novel multi-scale distance-weighted loss to boost segmentation accuracy near fracture sites, incorporating deep supervision and a smooth transition strategy during training to elevate local accuracy without compromising the overall performance.
- We curated a benchmark dataset of pelvic fracture CT images, encompassing 150 fractured cases with well-annotated ground-truth anatomical and fracture labels.

Our dataset and source code have been made publicly available at https://github.com/YzzLiu/FracSegNet.

## 2 Related work

### 2.1 Medical image segmentation

The encoder-decoder architecture introduced by U-Net has established a strong foundation for both 2D and 3D medical image segmentation tasks (9), (10). Subsequent models such as U-Net++ and V-Net have refined this approach with improvements like nested skip connections and volumetric convolutions (11), (12). More recently, researchers have enhanced U-Net by integrating new architectural concepts. Transformer-based hybrids, including Swin-UNETR and TransUNet, incorporate global context through self-attention mechanisms, while CNN-focused enhancements such as MedNext and STU-Net improve feature extraction using advanced convolutional techniques (13–16). Additionally, innovative models like U-Mamba employ state-space models to better capture long-range dependencies (17). Furthermore, systematic benchmarking, as demonstrated by nn-UNet, reveals that careful attention to implementation details, such as the choice of loss function and data augmentation strategies, can yield performance gains that rival or even surpass those achieved by novel architectural designs (18).

### 2.2 Bone segmentation

Bone segmentation methods can be broadly categorized into traditional approaches based on intensity, template-based methods, and deep learning-based methods (19–21). Traditional intensity-based approaches often struggle with the distinct intensity discrepancies between cortical and trabecular bones, compounded by the overlapping intensity ranges between trabecular bones and soft tissues. This often results in the formation of hollows within the segmentation masks. Such inaccuracies are particularly problematic in tasks like screw fixation planning, where a precise understanding of the pelvic bone topology is critical (19). Template-based methods involve registering a CT scan with a healthy template and employing graph partitioning techniques to propagate labels. However, this strategy heavily relies on the accuracy of registration and can yield unreliable results in the presence of fractures (20). Deep learning-based bone segmentation has demonstrated significant success across various anatomies, such as the pelvis, ribs, spine, and skull (22–25). Liu et al. applied a cascade 3D UNet for the anatomical segmentation of the hipbone, sacrum, and lumbar vertebrae in CT, demonstrating the effectiveness and robustness of deep learning methods in pelvic bone segmentation (22).

### 2.3 Fracture detection

The application of deep learning in handling fractured images was initially explored in fracture detection tasks aimed to facilitate diagnosis. It has been applied across various anatomical sites, including the hand, ribs, pelvis, and spine (26, 27), (28, 29). Notably, Jin et al. formulated rib fracture detection as a segmentation task. While this provided a rough outline of the fractured region, it is not capable of delineating the fracture surface and the fragment accurately (27). In the context of pelvic fractures, Ukai et al. integrates parallel 2D YOLOv3 models to detect pelvic fractures and subsequently combines 2D fracture candidate points to delineate the 2D fracture region (28). Additionally, Zeng et al. propose a two-stage structure-focused contrastive learning strategy that effectively exploits the symmetry of pelvic structures for fracture detection (30). While these methods can provide substantial aid in trauma diagnosis and clinical decision-making, they fall short in applications requiring precise delineation of fragments for image-guided surgery.

### 2.4 Fracture segmentation

Various methods have been explored to isolate fractured bone fragments from CT scans, including fixed or adaptive thresholding, watershed algorithms, non-rigid registration, sheetness-based approaches, and region growing (6, 31–34). These techniques generally rely on the intensity similarity and continuity of boundary gradients to segment fractures. For instance, Yuan et al. proposed a semi-automatic graph cut method based on continuous max-flow to segment pelvic fractures, which involves manual selection of seed points and a trial-and-error process (5, 35). Similarly, Wang et al. developed an automatic max-flow segmentation approach using graph cuts and boundary-enhancing filters. While effective in separating fragments, this method often struggles with fragments in collision or compression (36). Despite these advancements, a fully automatic and robust solution for fracture segmentation remains elusive.

Deep learning fracture segmentation remains a relatively under-explored area, yet several studies have demonstrated its significant potential. For instance, Yang et al. applied a two-stage Mask R-CNN model to locate and segment intertrochanteric fractures in 2D images (37). Kim et al. leverages a DeepLab model to automatically segment bone fragments in tibia and fibula from CT scans (38). Furthermore, Wang et al. employed the V-net architecture for segmenting intertrochanteric femoral fractures (39). Data size has been identified as a common challenge in these studies that limits the segmentation accuracy, especially for small bone fragments. This limitation underscores the need for innovative solutions in both the development of robust datasets and the more efficient use of available data.

### 2.5 Differences from the conference version

This study expands upon our initial conference paper presented at the 26th International Conference on Medical Image Computing and Computer Assisted Intervention (MICCAI 2023), advancing the original work in its performance, depth, and practical utility (40). Firstly, we have enhanced the fractured CT dataset with a larger patient cohort, a more comprehensive range of fracture types, and refined annotations of pelvic bone fragments. Secondly, we have optimized the design of the anatomical segmentation network and experimented on its training setups, substantially boosting its performance on fractured data. Thirdly, we have incorporated more thorough experiments on comparing methods and parameter searching for the fracture segmentation network, demonstrating the model's effectiveness and robustness with detailed analysis.

## 3 Methods

### 3.1 Overview

**Figure 1:**
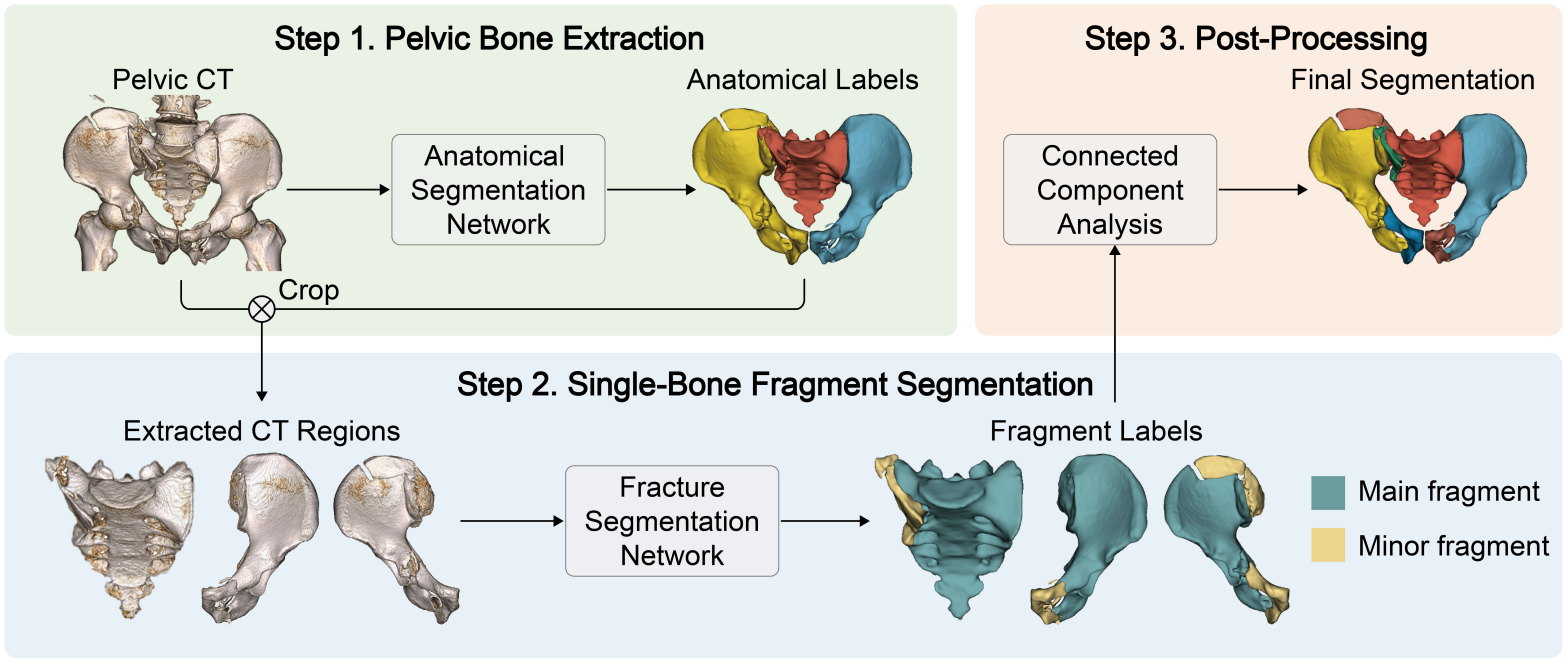
Overview of the proposed pelvic fracture segmentation method. In Step 1, a cascaded UNet is employed to predict anatomical labels from pelvic CT images, which are then utilized to extract the pelvic bones from the CT. In Step 2, a distance-weighted UNet is used to segment main and minor fragments from the extracted bone regions. In Step 3, connected component analysis is performed to obtain the final segmentation results.

Our study is dedicated to the automated segmentation of target bone fragments (specifically, the left and right hipbones and the sacrum) from CT scans. As shown in Figure 1, our methodology unfolds in three steps. Initially, an anatomical segmentation network, leveraging a cascaded 3D nn-UNet architecture, is deployed to isolate the pelvic bones from the CT scans. This network, pre-trained on a comprehensive dataset of pelvic CT images (22) undergoes further refinement on our dataset of fractured cases. Following this, a fracture segmentation network is used to segment the bone fragments within each extracted hipbone and sacral region. To establish a uniform labeling protocol across all fracture types, we assign three labels per bone: the background, the main fragment, and minor fragments. The main fragment, typically the most substantial piece located centrally within each bone, contrasts with the minor fragments, which represent the remainder. The post-processing step further separates and labels isolated components to yield the final segmentation result.

### 3.2 Pelvic bone extraction

In the initial step, we develop an anatomical segmentation network to extract pelvic bones from CT images. We employed a cascaded 3D UNet framework to predict anatomical labels from pre-processed CT images. Two five-layer UNet models are trained sequentially: The first UNet is trained on low-resolution images to enhance its contextual understanding through larger receptive field, producing coarse segmentations of hipbones and sacrum. Then, the second UNet is trained on full-resolution images to refine local details, taking concatenated coarse segmentation labels and CT volumes as inputs to produce precise segmentations.

The networks are pre-trained on the *CTPelvic1K* dataset, which contains over 1,000 high-resolution scans of pelvis, and is refined on the curated *Pelvic Bone Fragments with Injuries (PENGWIN)* dataset, which contains a broader range of fracture cases (detailed in Section 3.5).

### 3.3 Bone fragment segmentation

**Figure 2:**
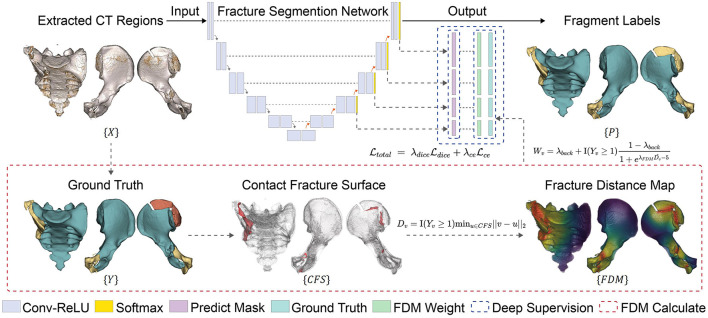
Training process of the fracture segmentation network. The contact fracture surfaces (CFS) are computed from the manually annotated ground truth to generate the fracture distance map (FDM), which is then mapped to the FDM weight and incorporated into the loss function. Deep supervision and the smooth transition strategy are employed to prevent excessive focus on local features.

We develop a fracture segmentation network to further isolate main and minor fragments from each extracted region. As shown in Figure 2, a 3D UNet, based on nn-UNet, is selected as the backbone model (18). The model learns a non-linear mapping relationship *M*:*X*→*Y*, where *X* and *Y* are the masked CT volume and ground truth fragment label, respectively.

#### 3.3.1 Fracture distance map

The contact fracture surface (CFS) is the part where the bones collide and overlap due to compression, and is the most challenging part for both human operators and network models to delineate. We are particularly concerned about the segmentation performance in this region. To this end, we introduce guidance into the network training using fracture distance map (FDM).

The FDM is computed on the ground-truth segmentation of each data sample before training. This representation provides information of the boundary, shape, and position of the object to be segmented. First, CFS regions are identified by comparing the labels within each voxel's neighborhood. Then, the distance of each foreground voxel to the nearest CFS is calculated as its distance value *D*_*v*_, and is then normalized.


(1) $$\begin{array}{l} D_{v}=\text{I}\left(Y_{v}\geq 1\right)\min_{u\in CFS}||v-u|{|}_{2}, \end{array}$$

(2) $$\begin{array}{l} \hat{D_{v}}=\frac{D_{v}}{\max_{v\in V}D_{v}}, \end{array}$$

where *V* is the set of all foreground voxels, *v* = (*h*_*v*_, *w*_*v*_, *d*_*v*_) is the index of a voxel in *V*, *u* = (*h*_*u*_, *w*_*u*_, *d*_*u*_) is the voxel index. *Y* is the ground-truth segmentation, I(*Y*_*v*_≥1) is the indicator function for foreground, and $\hat{D}$ is the normalized distance. The distance is then used to calculate the FDM weight *Ŵ*:


(3) $$\begin{array}{l} W_{v}=\lambda_{back}+\text{I}\left(Y_{v}\geq 1\right)\frac{1-\lambda_{back}}{1+e^{\lambda_{FDM}\hat{D_{v}}-5}}, \end{array}$$

(4) $${\hat{W}}_{v}=\frac{W_{v}\cdot |V|}{\sum_{v\in V}W_{v}}.$$

where λ_*back*_ is the weight for the background voxels, λ_*FDM*_ is the slope parameter in the activation function. To ensure the equivalence of the loss among different samples, the weights are normalized by their sum.

#### 3.3.2 Distance-weighted loss

The FDM weight Ŵ is then used to calculate the weighted Dice $L_{dice}$ and cross-entropy loss $L_{ce}$, so that the CFS gains more importance in training.


(5) $$\begin{array}{l} L_{dice}=1-\frac{2}{\left|L\right|}\underset{l\in L}{\sum }\frac{\underset{v\in V}{\sum }{\hat{W}}_{v}P_{v}^{l}Y_{v}^{l}}{\underset{v\in V}{\sum }{\hat{W}}_{v}P_{v}^{l}+\underset{v\in V}{\sum }{\hat{W}}_{v}Y_{v}^{l}}, \end{array}$$

(6) $$\begin{array}{l} L_{ce}=-\frac{1}{\left|V||L\right|}\underset{v\in V}{\sum }\underset{l\in L}{\sum }{\hat{W}}_{v}Y_{v}^{l}log\left(P_{v}^{l}\right), \end{array}$$

where *L* is the number of classes, $P_{v}^{l}$ and $Y_{v}^{l}$ are the output prediction and the ground truth for the *v*^*th*^ voxel of the *l*^*th*^ label. The overall loss is their weighted sum:


(7) $$\begin{array}{l} L_{total}\;=\;{\lambda_{dice}L}_{dice}+{\lambda_{ce}L}_{ce}, \end{array}$$

where λ_*dice*_ and λ_*ce*_ are balancing weights.

#### 3.3.3 Multi-scale deep supervision

We use a multi-scale deep supervision strategy in model training to learn different features more effectively (41). The deep layers mainly capture the global features with shape/structural information, whereas the shallow layers focus more on local features that help delineate fracture surfaces. Auxiliary losses are integrated into the decoder at different resolution levels (except the lowest resolution level). The losses are calculated using the corresponding down-sampled FDM ${Ŵ}_{v}^{n}$, and down-sampled ground truth $Y_{v}^{n}$. The loss for the *n*^*th*^ level $L^{n}$ is calculated with a different λ_*FDM*_ in Equation 3. The λ_*FDM*_ of each layer decreases by a factor of 2 as the depth increases, i.e., λ^*n*+1^ = λ^*n*^/2. In this way, the local CFS information are assigned more attention in the shallow layers, while the weights become more uniform in the deep layers.

#### 3.3.4 Smooth transition

To stabilize network training, we use a smooth transition strategy to maintain the model's attention on global features at the early stage of training and gradually shift the attention toward the fracture site as the model evolves (42). The smooth transition dynamically adjusts the proportion of the FDM in the overall weight matrix based on the number of training iterations. The dynamic weight is calculated using the following formula:


(8) $$W_{st}=\{\begin{array}{ll} J, & \text{if }t<\tau_{begin},\\ \frac{1}{1+\delta }J+\frac{\delta }{1+\delta }\hat{W}, & \text{if }\tau_{begin}\leq t\leq \tau_{begin}+\tau_{smooth},\\ \hat{W}, & \text{if }t>\tau_{begin}+\tau_{smooth}, \end{array}$$

(9) $$\begin{array}{l} \delta =-\ln\left(1-\frac{t-\tau_{begin}}{\tau_{smooth}+\epsilon }\right), \end{array}$$

where J is an all-ones matrix with the same size as the input volume, *t* is the current iteration number, τ_*begin*_ is the iteration where the transition begins, τ_*smooth*_ is the duration of the smooth transition phase, and ϵ is a small positive constant. The dynamic weight *W*_*st*_ is adjusted by controlling the relative proportion of J and *Ŵ*.

### 3.4 Post-processing

Connected component analysis (CCA) has been widely used in segmentation (43). However, its direct application to fracture segmentation is often complicated due to the collision between fragments. Nevertheless, after the removal of the main central fragment, the minor fragments become naturally isolated. Therefore, in the post-processing step, we further isolate the remaining minor fragments by CCA. The isolated components are then assigned different labels. Additionally, we exclude any fragments smaller than 1 cm^3^, as they typically do not significantly impact the outcomes in robotic surgery contexts.

### 3.5 Dataset

#### 3.5.1 Data collection and distribution

We curated *PENGWIN*, a dataset of 150 CT scans representing a wide range of common pelvic fractures. These scans were obtained from patients who underwent pelvic reduction surgery between 2017 and 2023 at six medical centers: Beijing Jishuitan Hospital (JST), Foshan Hospital of TCM (FSHTCM), the First Bethune Hospital of Jilin University (JLUFH), the Third Bethune Hospital of Jilin University (JLUTH), Nanfang Hospital (NFH), and Tianjin Hospital (TJH). Imaging was performed using seven CT scanners, including the Toshiba Aquilion Prime, United Imaging uCT 550, Philips Brilliance 64, Siemens Sensation 64, Toshiba Aquilion One, Siemens Somatom Force, and GE Optima CT660. The retrospective use of these scans was approved by the respective institutional ethics committees.

The dataset includes patients aged 16 to 94 years, comprising 63 females and 87 males. The average voxel spacing is (0.83, 0.83, 0.89) mm, with typical image dimensions of approximately (488, 426, 323). To ensure a representative distribution, we incorporated five primary fracture types: pelvic ring dislocation (5 cases), unilateral hip fracture (54 cases), bilateral hip fractures (31 cases), sacral fracture (5 cases), and combined sacral and hip fractures (55 cases). For model development, stratified sampling allocated 120 cases for training and 30 for testing.

#### 3.5.2 Annotation

The dataset is processed by two experienced annotators and a senior expert. The inter-annotator variability between annotators was characterized by an Intersection over Union (IoU) of 0.984 and an Adjusted Rand Index (ARI) of 0.993. The data annotation was structured into a four-step workflow:

- **Initial automatic segmentation:** we employ a pre-trained segmentation network based on the nn-UNet framework to produce preliminary anatomical segmentations (22). This network was trained on *CTPelvic1K*, with the majority of them not presenting any fractures.
- **Manual refinement of anatomical labels:** The initial anatomical labels undergo a meticulous refinement process by annotators using the 3D Slicer platform.
- **Identification of fractured fragments:** leveraging the refined anatomical labels, the annotators identify and label fractured bone fragments. This operation is also carried out on the 3D Slicer platform.
- **Expert validation:** as a final checkpoint, a senior expert rigorously reviews and modifies the annotated fracture labels, ensuring their precision and consistency.

#### 3.5.3 Labeling rule

The primary objective of our investigation is to streamline the process for automated fracture reduction planning in robotic surgeries. Within this framework, the main fragment is maneuvered to a predefined location using a robotic arm, while the minor fragments are either manually adjusted by surgeons or simply ignored. Based on our findings, separating the minor fragments is often not necessary. Hence, for consistency in annotations across our dataset, we limit the fragment count for each bone to three. This rule has been uniformly applied across all 150 cases within our dataset. In addition, to enhance the utility of our research for future studies, we have compiled a separate dataset version that includes detailed separation of minor fragments.

## 4 Experiments and results

### 4.1 Implementation

The method was implemented with PyTorch and SimpleITK. Experiments were performed with an Intel Xeon 40-core CPU, a Quadro RTX 5000 GPU, and a 256 GB memory.

#### 4.1.1 Anatomical segmentation network training

All images underwent b-spline interpolation to resample voxel spacing to (0.83, 0.83, 0.89) mm, followed by z-score normalization. To enhance the variability of our dataset, for each training sample, four augmented samples are generated. These images were created by applying random elastic distortions within a range of 80%–120%, along with random translations and rotations within the ranges of -20 to 20 mm and -30 to 30 degrees for each axis, respectively. To further strengthen resilience against noise, random noise was added with a probability of 15%. This included Gaussian blur values ranging from 0.5 to 1.0, brightness scaling from 75% to 125%, contrast adjustments from 75% to 125%, and gamma transformations from 0.7 to 1.5.

ADAM optimizer with an initial learning rate of 0.0001 and a batch size of 2 was used. The learning rate was subjected to exponential decay. We performed five-fold cross-validation on training set. Each model underwent training for 2,000 epochs.

#### 4.1.2 Fracture segmentation network training

We cropped the resampled bone volumes by calculating bounding box, and normalized them with z-score. For each training sample, eight augmented images were generated. This process involved mirror flipping along three axes, accompanied with random distortions, translations, rotations, and noise simulation similar to those employed in the anatomical network.

ADAM optimizer with a learning rate of 0.0001 and a batch size of 2 was used. λ_*back*_ was set 0.2. Both λ_*dice*_ and λ_*ce*_ were set to 1. The initial λ_*FDM*_ was set to 16. We conducted a five-fold cross-validation on the training set, where each model underwent training for 2000 epochs.

### 4.2 Evaluation

We assessed the performance of various methods in anatomical and fracture segmentation using Dice Similarity Coefficient (DSC), average symmetric surface distance (ASSD), and the 95th percentile of the Hausdorff Distance (HD_95_). To account for labels that were entirely missing in the prediction, their HD_95_ and ASSD were assigned the diameter and radius of the ground truth's circumferential sphere, respectively. Furthermore, we also incorporated the median HD_95_ for a more comprehensive evaluation, which mitigates the impact from the failure cases. For fracture segmentation, we evaluated the local Dice similarity coefficient (LDSC) within a 10 mm range around the CFS to measure performance in critical areas. Two-tailed t-tests were used to examine the statistical significance.

### 4.3 Experiments on anatomical segmentation

We conducted a comparative analysis of anatomical segmentation models trained on three distinct datasets: *CTpelvic1K* alone, *PENGWIN* alone, and a combination where training began on *CTpelvic1K* followed by fine-tuning on *PENGWIN*.

**Figure 3:**
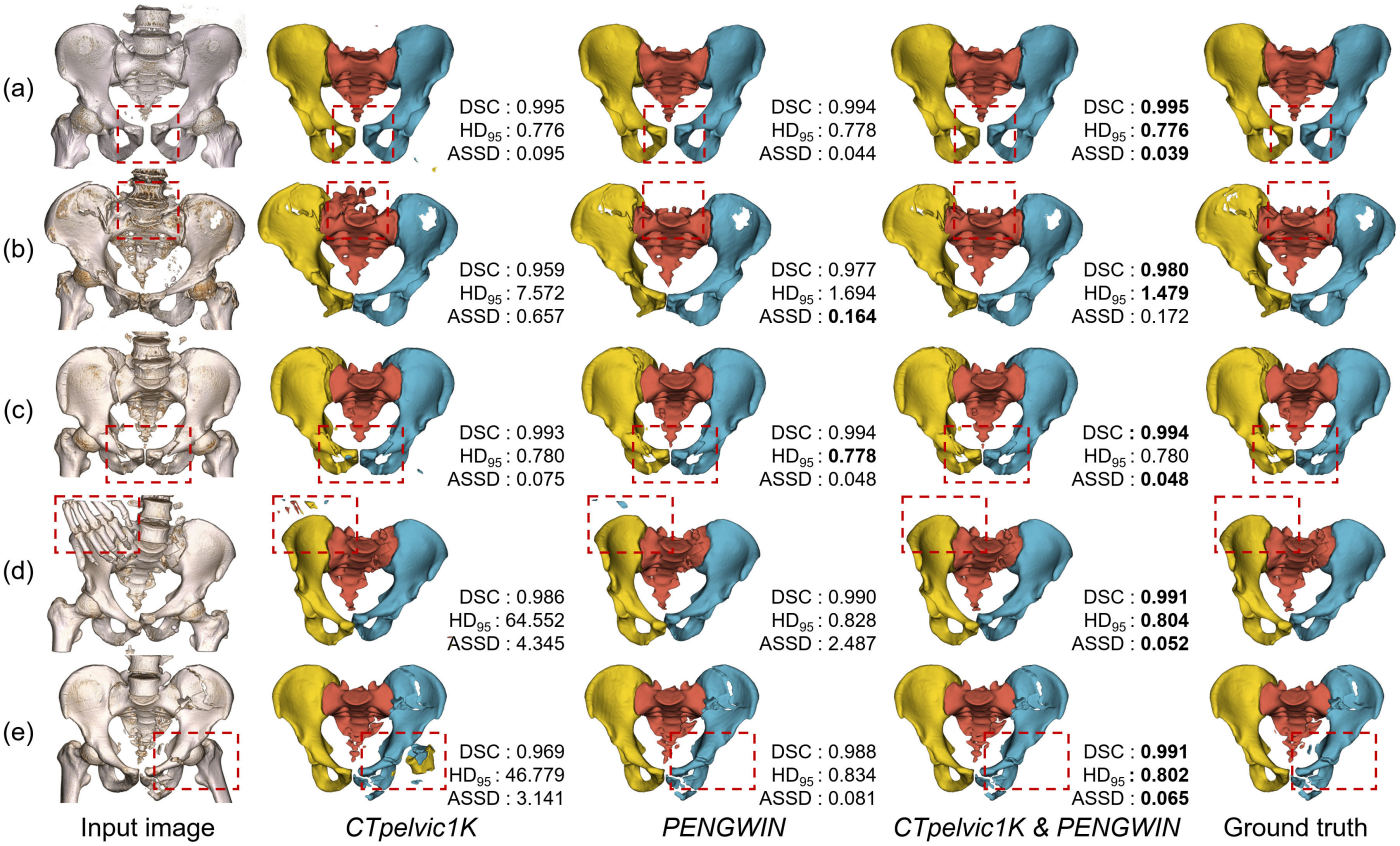
Example anatomical segmentation results from different models. **(a)** Pelvic ring dislocation. **(b)** Unilateral hip fracture. **(c)** Bilateral hip fractures. **(d)** Sacral fracture. **(e)** Combined sacral and hip fractures.

Figure 3 illustrates the results across five typical fracture types. The model trained solely on *CTpelvic1K* displayed suboptimal performance, particularly in cases involving lumbar sacralization and fractures with fragments distanced from the main fragment. This limitation is largely attributed to the dataset's predominance of intact pelvis scans and a limited diversity in fracture types. In contrast, the model trained on the combined dataset exhibited superior overall performance, benefiting significantly from the enhanced variety in fracture characteristics. Table 1 provides a quantitative comparison of the anatomical segmentation results on the test set, with paired t-tests indicating that the model trained on the combined dataset achieved better or at least comparable results to other methods across all evaluated metrics.

**Table 1:** Quantitative comparison of anatomical segmentation.

**Training data**	**Metric**	**Sacrum**	**Left hipbone**	**Right hipbone**	**Total**
*CTpelvic1K*	DSC	0.982 ± 0.016*	0.985 ± 0.015*	0.983 ± 0.015*	0.983 ± 0.010*
	HD_95_ (mean)	1.595 ± 3.67	16.382 ± 41.580*	21.140 ± 58.755	13.039 ± 23.683*
	HD_95_ (median)	0.810	0.800*	0.800	0.835*
	ASSD	0.548 ± 0.934*	1.109 ± 2.242*	1.989 ± 6.06	1.215 ± 2.247*
*PENGWIN*	DSC	0.986 ± 0.007*	0.991 ± 0.004*	0.990 ± 0.005*	0.989 ± 0.004*
	HD_95_(mean)	0.946 ± 0.521	7.534 ± 36.835	**0.867** **±0.309**	3.115 ± 12.260
	HD_95_(median)	**0.801**	0.795	0.800	0.804
	ASSD	0.292 ± 0.610	0.374 ± 0.988	0.353 ± 0.816	0.340 ± 0.627*
*CTpelvic1K & PENGWIN*	DSC	**0.987** **±0.007**	**0.993** **±0.003**	**0.992** **±0.005**	**0.991** **±0.004**
	HD_95_(mean)	**0.916** **±0.387**	**0.804** **±0.055**	0.867 ± 0.340	**0.862** **±0.185**
	HD_95_(median)	0.802	**0.793**	**0.798**	**0.802**
	ASSD	**0.098** **±0.079**	**0.080** **±0.106**	**0.062** **±0.042**	**0.080** **±0.053**

HD_95_ and ASSD are reported in mm. The best values are shown in bold. Statistically significant differences compared to the last method are indicated by *p < 0.05.

### 4.4 Experiments on fracture segmentation

#### 4.4.1 Ablation study and benchmark comparison

We conducted an ablation study for the fracture segmentation network, comparing the proposed method (FDMSS-UNet) against the model without smooth transition and deep supervision (FDM-UNet) and the model without distance weighting (UNet). In addition, we also compared the methods against the traditional max-flow segmentation approach and a Swin-UNETR model (5, 13).

**Figure 4:**
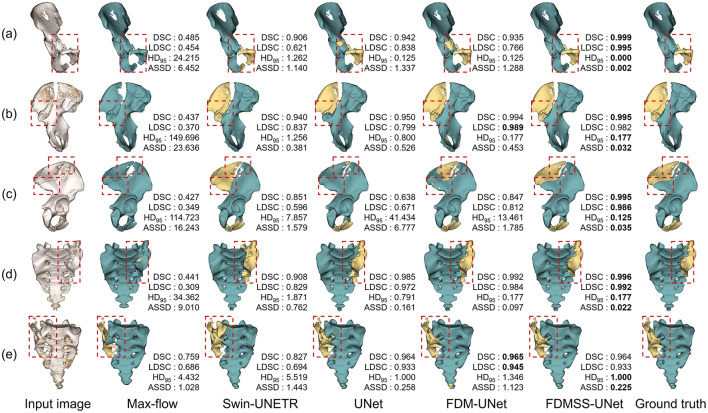
Example fracture segmentation results using different methods. **(a)** Anterior ring fracture. **(b)** Posterior ring fracture. **(c)** Combined fracture of the anterior and posterior ring. **(d)** Left sacral fracture. **(e)** Right sacral fracture. Fractured regions are marked with red boxes.

**Figure 5:**
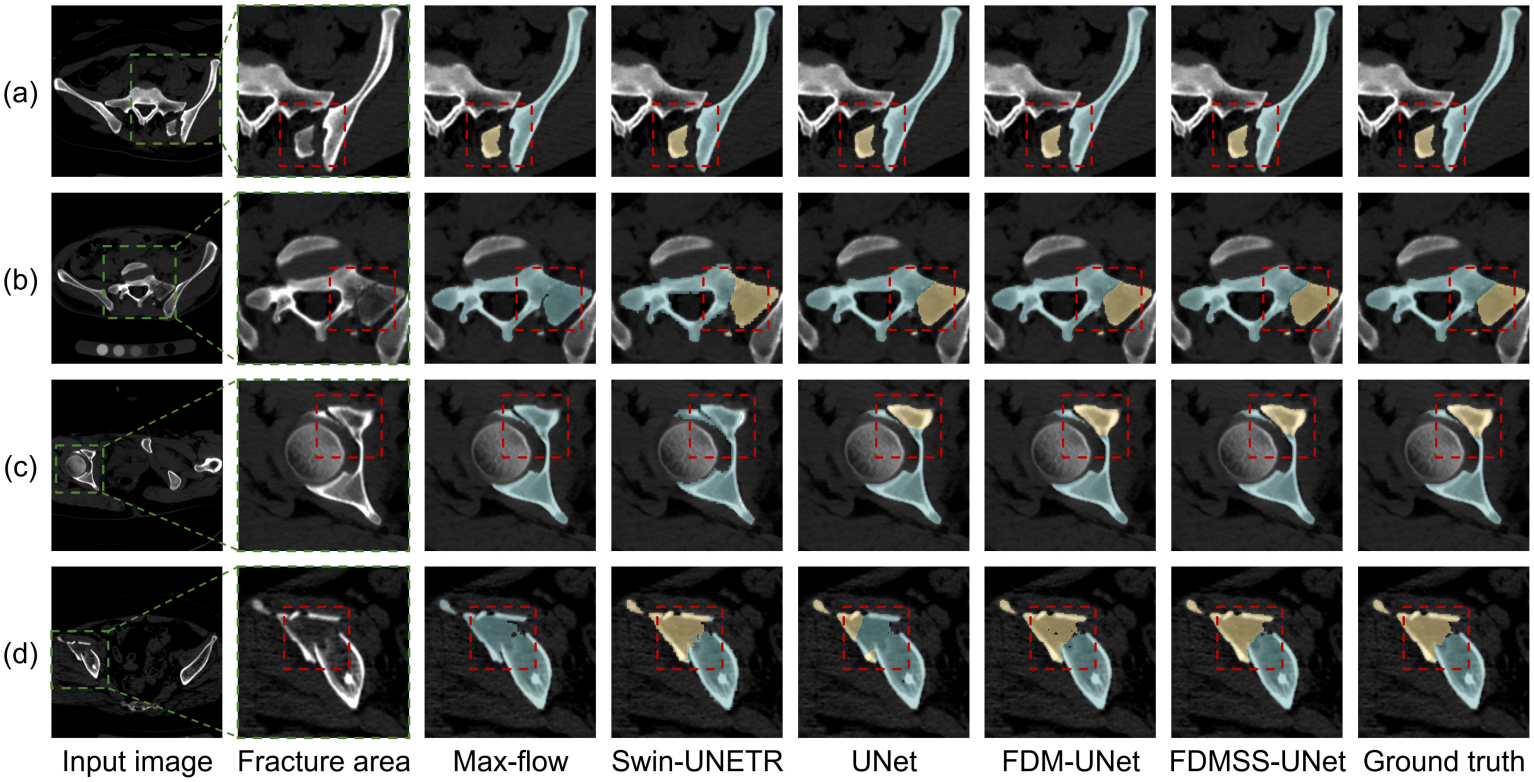
Example fracture segmentation results shown on 2D axial slices. **(a)** Isolated and displaced fragments. **(b)** Isolated but stable fragments. **(c)** Partially separated fragments. **(d)** Compressed and colliding fragments. Fractured regions are marked with red boxes.

**Figure 6:**
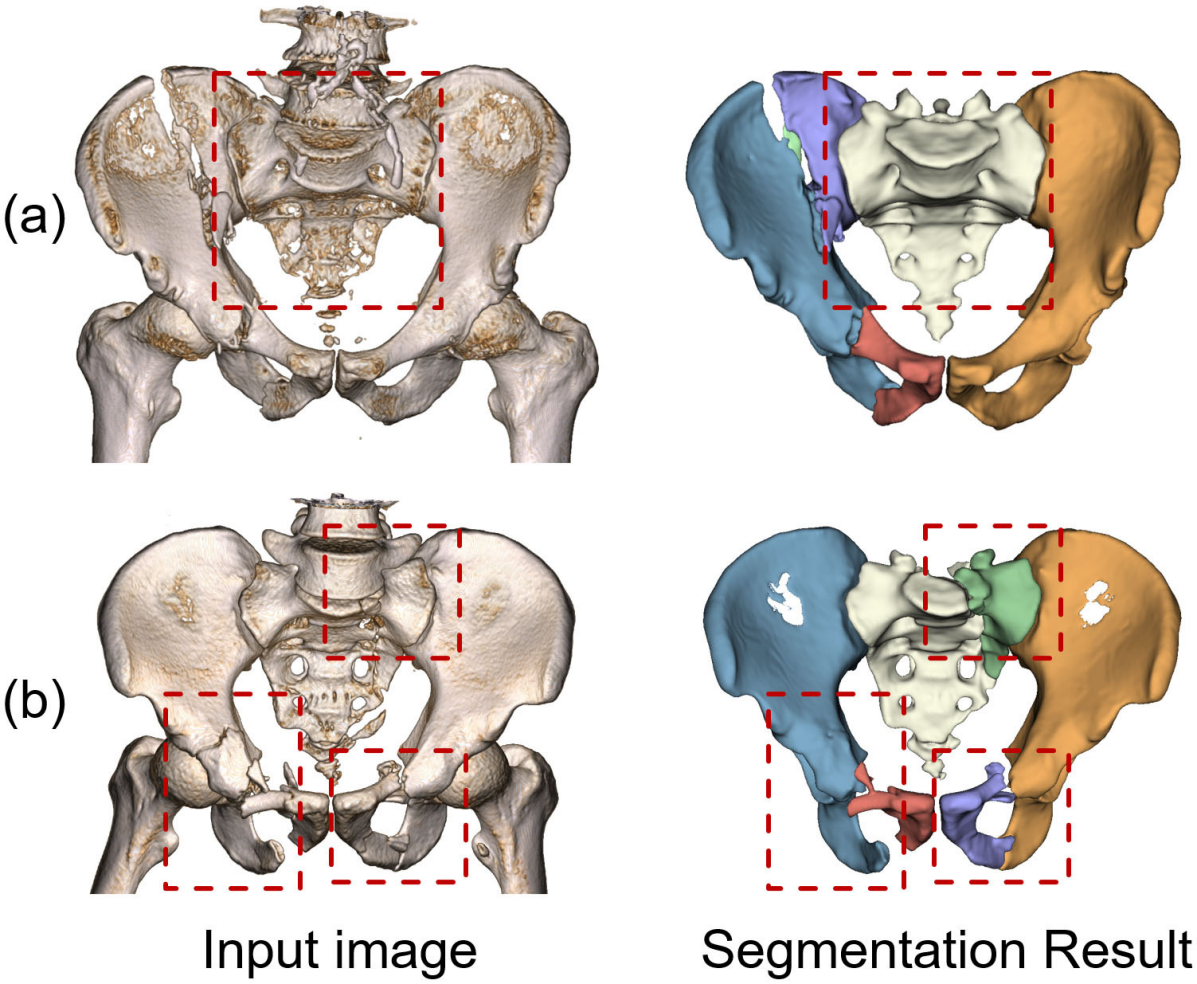
Fracture segmentation results on **(a)** osteoporotic, and **(b)** a highly complex case.

Figures 4, 5 provide qualitative comparisons in both 3D and 2D slice views, respectively. While max-flow segmentation yields reasonable results in cases where the CFS is clear or the fragments are non-contacting, it underperforms in more complex scenarios. Both Swin-UNETR and UNet effectively identify fracture fragments but struggle with accurate delineation in complex CFS areas, leading to errors in fracture surface identification. FDM-UNet improves upon max-flow, Swin-UNETR, and UNet near the CFS areas but occasionally misidentifies non-fractured areas far from the CFS as fractured. The inclusion of FDM weighting and deep supervision with a smooth transition in FDMSS-UNet significantly enhances its performance, particularly near the CFS, making it the most effective model among those tested. In addition, Figure 6 presents the segmentation performance on both an osteoporotic fracture case and a highly complex fracture case, demonstrating that our method is effective for these two types of fracture cases.

Table 2 presents the quantitative results. The main fragments, which occupy a larger proportion and are always present, generally show better metric outcomes compared to the minor fragments. Deep learning methods significantly outperform traditional max-flow in the success rate of identifying fragments, particularly small ones, with statistically significant improvements. The introduction of FDM notably increases prediction accuracy in the CFS area. The strategies of deep supervision and smooth transition stabilize training, balance local and global performance, and yield the best overall results.

**Table 2:** Quantitative comparisons of fracture segmentation.

**Method**	**Metric**	**Hip-main**	**Hip-minor**	**Sacral-main**	**Sacral-minor**	**All**
**Max-flow**	DSC	0.946 ± 0.053*	0.272 ± 0.396*	0.929 ± 0.150*	0.235 ± 0.320*	0.765 ± 0.251*
	LDSC	0.765 ± 0.087*	0.145 ± 0.298*	0.723 ± 0.110*	0.166 ± 0.256*	0.453 ± 0.180*
	HD_95_	16.782 ± 20.792*	126.800 ± 100.140*	5.807 ± 9.317*	71.083 ± 59.986*	34.606 ± 50.730*
	ASSD	1.372 ± 1.898*	27.049 ± 21.791*	0.691 ± 1.156*	14.081 ± 10.993*	7.132 ± 10.091*
**swin-UNETR**	DSC	0.944 ± 0.016*	0.812 ± 0.193*	0.939 ± 0.023*	0.854 ± 0.097*	0.911 ± 0.077*
	LDSC	0.463 ± 0.156*	0.565 ± 0.194*	0.543 ± 0.096*	0.725 ± 0.069*	0.541 ± 0.132*
	HD_95_	4.156 ± 8.274*	18.850 ± 20.617*	1.699 ± 1.863*	10.581 ± 13.652	3.195 ± 6.632*
	ASSD	0.557 ± 0.445*	2.880 ± 4.403*	0.392 ± 0.192*	1.482 ± 2.106*	1.035 ± 1.681*
**UNet**	DSC	0.994 ± 0.019	0.944 ± 0.151*	0.990 ± 0.031	0.940 ± 0.094	0.981 ± 0.062*
	LDSC	0.946 ± 0.052*	0.917 ± 0.088*	0.925 ± 0.034*	0.918 ± 0.040	0.929 ± 0.063*
	HD_95_	2.484 ± 11.517	7.275 ± 23.304*	1.209 ± 4.494	7.367 ± 13.929	1.887 ± 7.776*
	ASSD	0.176 ± 0.798	0.959 ± 2.657	0.093 ± 0.312	0.929 ± 2.053	0.331 ± 1.159*
**FDM-UNet**	DSC	**0.997** **±0.010**	0.955 ± 0.133	0.990 ± 0.034	0.938 ± 0.100	0.984 ± 0.054
	LDSC	0.957 ± 0.043	0.930 ± 0.083	0.921 ± 0.035*	0.913 ± 0.043	0.938 ± 0.058
	HD_95_	1.091 ± 5.277	5.722 ± 15.094	0.986 ± 3.489	7.415 ± 13.786	**1.154** **±5.481**
	ASSD	0.077 ± 0.305	1.139 ± 3.503	0.080 ± 0.259	1.132 ± 2.206*	0.346 ± 1.290*
	DSC	0.996 ± 0.009	0.950 ± 0.128	0.993 ± 0.015	0.950 ± 0.057*	0.984 ± 0.048
**FDMSS-UNet**	LDSC	0.925 ± 0.166	0.914 ± 0.115*	0.915 ± 0.039*	0.899 ± 0.062	0.917 ± 0.124*
**(Three-class)**	HD_95_	**1.052** **±4.426**	10.150 ± 32.219*	0.569 ± 0.968	7.282 ± 13.032*	1.211 ± 5.092
	ASSD	**0.073** **±0.250**	1.529 ± 4.577	0.058 ± 0.115	0.864 ± 1.907	0.409 ± 1.599*
	DSC	0.994 ± 0.019	0.938 ± 0.155*	**0.996** **±0.005**	0.938 ± 0.111	0.981 ± 0.061
**FDMSS-UNet**	LDSC	0.945 ± 0.057*	0.907 ± 0.107*	**0.939** **±0.029**	**0.932** **±0.040**	0.928 ± 0.073
**(Separate data)**	HD_95_	2.403 ± 10.931	8.860 ± 24.888	**0.320** **±0.402**	12.290 ± 24.137	1.382 ± 6.204
	ASSD	0.169 ± 0.702	1.361 ± 4.118	**0.045** **±0.085**	0.795 ± 1.227	0.398 ± 1.492*
	DSC	0.996 ± 0.014	**0.957** **±0.146**	0.993 ± 0.014	**0.955** **±0.056**	**0.986** **±0.055**
**FDMSS-UNet**	LDSC	**0.958** **±0.042**	**0.932** **±0.077**	0.929 ± 0.036	0.920 ± 0.050	**0.940** **±0.056**
**(Mixed data)**	HD_95_	1.202 ± 5.883	**3.595** **±12.403**	0.548 ± 0.898	**6.325** **±12.351**	1.262 ± 5.811
	ASSD	0.082 ± 0.366	**0.719** **±2.445**	0.054 ± 0.101	**0.764** **±1.759**	**0.234** **±0.981**

HD_95_ and ASSD are presented in mm. The best values are shown in bold. Statistically significant differences compared to the last method are indicated by *p < 0.05.

#### 4.4.2 Comparison of training setups

We compared the performance of three different training setups for the FDMSS-UNet: (a) a three-class setup that differentiates the main fragment, anterior iliac (or left sacral) fragments, and posterior iliac (or right sacral) fragments; (b) a two-class setup where models were trained separately for the hipbone and sacrum data, each distinguishing between the main and minor fragments; and (c) a two-class setup with mixed hipbone and sacrum data used for training. The results are also shown in Table 2. Overall, the two-class models demonstrated superior performance across most metrics compared to the three-class model, with exceptions on HD_95_ and ASSD for the hipbone main fragment. Moreover, training a mixed model with both hipbone and sacrum data generally yielded better results than training separate models, likely due to a more diverse representation of fracture surface characteristics in the mixed dataset. While the separate sacrum model performed slightly better than the mixed model, the difference was not statistically significant.

#### 4.4.3 Hyper-parameters for smooth transition

To assess the behavior of the proposed smooth transition scheme, we conducted a grid search experiment to optimize τ_*begin*_ and τ_*smooth*_ in Equation 8, exploring values of 0, 500, and 1000 for each. The results indicate that the FDMSS-UNet achieved the best overall performance across most metrics when τ_*begin*_ is set to 0 and τ_*smooth*_ is set to 1000.

#### 4.4.4 Influence of fragment size

**Figure 7:**
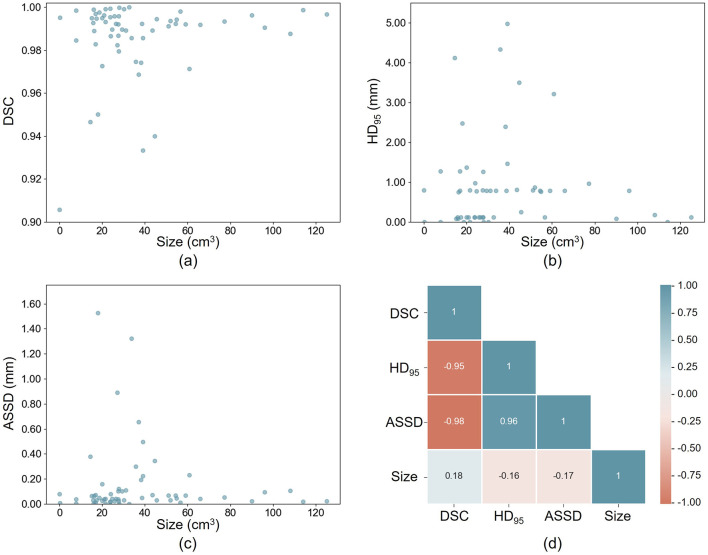
Influence of fragment size on segmentation accuracy. **(a–c)** the relationship between fragment size and DSC, HD_95_, and ASSD. **(d)** the correlation matrix between size and the metrics.

We evaluated the impact of fragment size on segmentation accuracy using the FDMSS-UNet. The results, shown in Figure 7, reveal no significant correlation between the size of the fragments and the overall segmentation accuracy. Specifically, DSC exhibited a weak positive correlation with fragment size, while HD_95_ and ASSD exhibited a weak negative correlation with fragment size.

## 5 Discussion

### 5.1 Effectiveness of the distance-weighted loss

Our network is trained using a FDM-based loss function and multi-scale deep supervision and smooth transition strategies. Compared to other methods, our approach utilizes FDM related to the CFS to guide network training, helping to focus on features near the CFS. Multi-scale deep supervision and smooth transition ensure local accuracy improvements without affecting overall performance. Experimental results show that our method achieves the best results.

In practical applications, especially in semi-automatic pipelines where human operators can modify and refine network predictions, accurate initial segmentation near the fracture site is highly desirable. The fracture surface itself is often complex and intertwined, making it difficult for manual operations. Our method can accurately predict main and minor fragments, greatly simplifying the workflow. Even when network predictions are inaccurate, manual operations on a 3D view can suffice for quick modifications in most cases, eliminating the need for inefficient slice-by-slice handcrafting.

### 5.2 Potential impacts on subsequent tasks

In addition to improving efficiency, our method significantly enhances the delineation of fracture surfaces and ensures a consistently filled bone region without gaps in the marrow areas, compared to traditional max-flow segmentation techniques used in commercial softwares. These improvements are crucial for various subsequent tasks in image-guided reduction surgery (44).

First, precise segmentation of bone fragments and fracture surfaces facilitates accurate alignment, enabling accurate target pose planning and navigation. It also minimizes interference in collision detection, reducing the risk of unexpected tool-bone contact during intraoperative navigation. Errors in segmentation can lead to misjudgments, increasing surgical complications. By providing a complete and reliable bone model, our approach enhances surgical safety. Furthermore, an intact bone mask is essential for precise screw placement planning. Our method ensures structural continuity, allowing for accurate and safe trajectory design, thereby reducing the risks of implant failure and neurovascular injury (45). Additionally, our approach improves intraoperative image registration by eliminating undesired inner surface points (46). Conventional methods struggle to differentiate trabecular and cortical bone boundaries, leading to registration errors. By enhancing segmentation accuracy, our method improves point cloud registration, ensuring precise alignment between preoperative CT and intraoperative CBCT models. This, in turn, enhances the reliability of surgical guidance.

### 5.3 Limitations and future work

**Figure 8:**
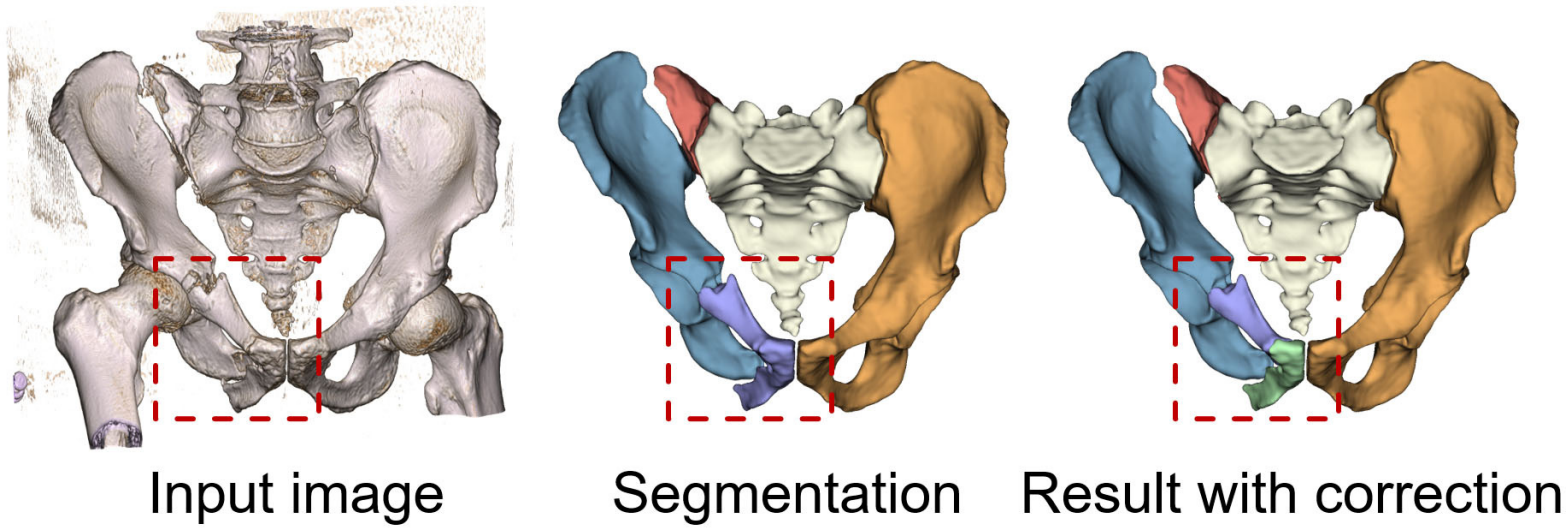
Failure segmentation examples on specific fractures that are not completely separated but have undergone significant distortion.

The variability in the number of bone fragments across different cases presents a challenge for deep learning-based segmentation approaches. As mentioned in Sec. 3.5, our study addresses this by implementing a consistent labeling strategy that simplifies the annotation process and ensures uniformity across the dataset. We limit the number of fragments for each bone to three, which aligns with the requirements of automatic fracture reduction planning for robotic surgery (47). While this approach streamlines the labeling process and reduces the complexity of the segmentation task, it is possible that the CCA cannot fully isolate the smaller fragments. Figure 8 presents an example of segmentation failures in case where fractures, though not completely separated, have experienced significant distortion. These situations complicate fracture delineation, occasionally resulting in imprecise segmentation. However, with minimal manual adjustments, the resulting segmentations remain suitable for subsequent tasks. Furthermore, the current study was conducted on a limited benchmarks and did not incorporate validation on additional external datasets. Due to the difficulty of sourcing additional large dataset with pelvic fracture, which is rare due to its low incidence rate, we resort to further validating the robustness of the proposed dataset and method using a few external special cases (Figure 6).

In future work, we plan to investigate instance segmentation setup that accommodates arbitrary number of fragment labels, potentially offering a more detailed representation of fracture cases. In addition, we also plan to simplify the current framework by replacing the initial anatomical segmentation network with a more lightweight bounding box detection network, which could potentially accelerate the process, as well as to prevent error accumulation across segmentation modules. Furthermore, we aim to extend our evaluations by including a broader range of benchmarks and datasets, thereby enhancing the generalizability of our findings. We also plan to apply the proposed method into downstream tasks including automatic target pose planning and CT-CBCT image registration to validate its clinical feasibility in the context of robot-assisted reduction surgery (46), (48), (49). We plan to assess the performance through retrospective case studies, cadaver experiments, and clinical experiments.

## 6 Conclusion

We have proposed an automatic segmentation approach for pelvic fractures utilizing deep convolutional networks, which accurately isolates bone fragments in CT scans. Our approach incorporates a multi-scale distance-weighted loss and deep supervision with a smooth transition strategy, significantly enhancing segmentation precision at fracture sites while maintaining robust overall performance. We have evaluated our method on a well-annotated benchmark dataset of 150 pelvic fracture CT scans, which has been made publicly available to foster further research in this field. The experimental results demonstrate a significant improvement over traditional max-flow method and state-of-the-art network model. The proposed method holds promise for improving image-guided surgeries through enhanced surgical planning, registration, and navigation, ultimately contributing to better clinical outcomes.

## Data Availability

The datasets presented in this study can be found in online repositories. The names of the repository/repositories and accession number(s) can be found below: https://github.com/YzzLiu/FracSegNet.
